# Organizational Culture and Trust Affect the Team-BasedPractice and Job Satisfaction of Nurse Practitioners in Acute Care Hospitals: A National Survey

**DOI:** 10.1155/2024/2049627

**Published:** 2024-01-16

**Authors:** Sheng-Shiung Huang, Hsiu-Fang Jen, Shiow-Luan Tsay, Ya-Jung Wang, Heng-Hsin Tung

**Affiliations:** ^1^College of Nursing and Health Sciences, Da-Yeh University, Changhua, Taiwan; ^2^Department of General Surgery, China Medical University Beigang Hospital, Yunlin, Taiwan; ^3^Department of Nursing, Da-Yeh University, Changhua, Taiwan; ^4^School of Nursing, National Yang Ming Chiao Tung University, Taipei, Taiwan

## Abstract

**Background:**

The link between organizational culture, organizational trust, job satisfaction, and team-based practice among nurse practitioners (NPs) has not been examined simultaneously.

**Aim:**

To identify the effects of organizational culture, organizational trust, and other factors on NPs such as job satisfaction and team-based practice.

**Methods:**

We used a cross-sectional design with a national sample. Data were collected using an online survey of 1,100 NPs working in acute care settings. The survey included demographic and working characteristics, the Organizational Culture Scale, the Organizational Trust Scale, the Misener Nurse Practitioner Job Satisfaction Scale (MNPJSS), and the NP-physician relations (NP-PR) subscale of the Nurse Practitioner Primary Care Organizational Climate (NP-PCOCQ). Multiple regression analysis with a stepwise selection method explored potential factors that influence job satisfaction and team-based practice.

**Results:**

A learning environment, psychological safety, senior leadership support, commitment to the organization, and the organizational culture and trust were positively associated with higher job satisfaction, which accounted for 49.2% of the variance in NPs' job satisfaction. Organizational trust, commitment to the organization, and learning environment promoted better team-based practice significantly. Also, NPs working a fixed shift pattern showed higher levels of team-based practice. These factors accounted for 23.66% of variances in team-based practice.

**Conclusion:**

Organizational culture and organizational trust affect the job satisfaction and team-based practice of NPs in acute care practices. *Implications for Nursing Management*. Acute care hospitals are encouraged to develop policies to enhance a learning environment, a supportive organizational culture, and trust in NPs' practice.

## 1. Introduction

Within 3 years, Taiwan is entering a superaged society (20% of the population above 65 years old in 2026) [1].This will impose an even higher demand and burden on the healthcare system. Since 2006, in response to the growing demands of healthcare in the aging population, nurse practitioners (NPs) hold a key position as healthcare providers in acute care hospitals.

The scope of Nurse Practitioners' (NPs) practice was influenced by state laws and regulations in the USA, leading to three primary practice classifications: full practice, reduced practice, and restricted practice (American Association of Nurse Practitioners) [2]. Notably, NPs' practice scope in Taiwan aligns closely with the US model of restricted practice, involving supervision by physicians during clinical patient care [3].

Currently, more than 10,000 NPs provide healthcare in more than 350 acute care hospitals in Taiwan (Taiwan Association of Nurse Practitioners [4]. A recent study documented that NPs not only provide high-quality healthcare but also alleviate the burden on the healthcare system [5]. Hence, developing and retaining the NP workforce is a critical strategy for enhancing the quality and outcomes of acute care organizations. Specifically, the job satisfaction of NPs within acute care practices contributes to the care outcomes and retention of NPs [6].

NPs in Taiwan mainly work closely with physicians using a team-based practice model. A higher team climate or collaboration reported better care outcomes and higher job satisfaction [7]. NP studies further suggested that the various organizational supports enhance the job satisfaction of NPs [8–10]. For instance, findings from Ho et al. [8] indicated that NPs who perceived higher levels of organizational support experienced greater job satisfaction. Similarly, Luo et al. [9] concluded that healthcare administrators could enhance NPs' job satisfaction by improving organizational support in practice. In addition, studies on physicians similarly documented that organizational culture affects physicians' trust in the organization, which impacts job satisfaction and patients' trust [11, 12], especially when physicians perceive their organizational cultures to have an emphasis on quality, communication, and information, cohesiveness among clinicians, and values alignment between physicians and leaders [12]. However, those studies were conducted in the medical professions. Whether those results can be applied to NPs warrant further study to verify the results. Furthermore, the relationship between organizational culture, trust, team-based practice, and job satisfaction of NPs in acute care practices requires to be examined simultaneously. The results of the study can provide vital pieces of information to administrators for creating policies that could enhance team-based practice and job satisfaction of NPs.

Organizational culture is defined as the mutual perspective, assumptions, and standards of an organization's membership, and strong organizational cultures can support staff members to accomplish goals, complete tasks assigned to them, and perceive fulfillment in their job [13]. An enhanced organizational culture could also enable healthcare professionals to cope with complex and changing environments [14]. Several studies have shown that organizational culture is an important factor in job satisfaction [13, 15–17]. Zhang and Li [17] found a relationship between organizational culture and employee satisfaction. Kim et al. [18] reported that organizational culture significantly predicted job satisfaction among registered nurses. Similarly, Tsai [19] stated that organizational culture significantly increased job satisfaction in Taiwan. To summarize, the supportive organizational culture was beneficial for healthcare professionals to practice effectively, and hence they demonstrated better job satisfaction. Although a positive correlation between organizational culture and job satisfaction has been reported among healthcare professionals or nurses, the results of previous studies were limited by sample size, generalizability, and non-NP samples. The link between organizational culture and job satisfaction among NPs has yet to be explored.

Interdisciplinary teamwork is an essential model for delivering healthcare to patients in acute care settings. Teamwork or team-based practice is defined as activities or processes through which team members achieve common goals by collaborating as a team [20]. Healthcare practice required well communication and cooperation between diverse clinical professionals. The relationship between NPs and physicians is important in team-based practice, which promotes better health outcomes [21]. Moreover, a strong culture engaged healthcare professionals to develop stronger teams with competitive advantages [22]. Several studies have shown the effects of organizational culture on team-based practice. Clarke [23] indicated that organizational culture develops mutual learning that facilitated discussions and negotiations among team members to meet patients' needs and achieve interdisciplinary team working. Abu-Jarad et al. [24] noted that organizational culture is a key dimension in studies that investigate organizational performance, and they found that organizational culture was positively correlated with organizational performance. Körner et al. [25], in a multicenter study, also found a direct influence of organizational culture on teamwork. As this literature has shown, a supportive organizational culture was the benefit of establishing effective teamwork. However, these results were based upon data from a small sample, and the specific influence of different dimensions of organizational culture on team-based practice among NPs is still unclear.

Although the influence of organizational culture on job satisfaction or teamwork has been studied extensively, studies within acute care settings among NPs are still scarce. Moreover, a significant body of previous studies measured culture as a single dimension. Measuring only one dimension of culture may cause a narrow view of the concept that ignores various sources of heterogeneity such as the environment or management support. Yet, organizational culture is a multidimensional construct, so the influence of organizational culture on professional outcomes can be viewed from a broader perspective [26]. Therefore, it is necessary to explore the influence of multiple dimensions of culture on NP practice outcomes such as job satisfaction and team-based practice among NPs.

In addition, organizational trust was a critical factor for job satisfaction. Organizational trust could be viewed as the faith of employees that the organization will make an all-out effort to achieve the commitment of employees [27]. Moreover, organizational trust may enhance the cooperation between employees and organizations, lead to effective communication, and compensate for any inadequate abilities of employees [28, 29]. Evidence from management and nursing studies suggested that organizational trust was essential for well-function organizations [30–32]. However, only a few studies have investigated the correlation between organizational trust and job satisfaction with nurses or healthcare professionals. For example, a positive correlation between organizational trust and job satisfaction was supported for nurses [31] and physicians [30]. Furthermore, clinicians with improved or stable high trust reported higher satisfaction than those whose trust declined [11]. Nevertheless, the role and context of practice differed between NPs and other employees. At present, there are few studies focusing on the effects of organizational trust on job satisfaction among NPs.

Likewise, the correlation between organizational trust and teamwork was also examined by previous literature. Nonetheless, research on the influence of organizational trust on teamwork in the clinical field is scarce. Isik et al. [33] found that there is a positive relationship between teamwork and organizational trust among workers in call centers. Tekingündüz et al. [34] revealed that employees who worked at hospitals with higher levels of trust could feel valuable and important; hence, they were more willing to perform better. In addition, recent studies identified that organizational trust was positively related to team performance [35, 36]. However, these results were focused on a nonclinical sample, and the generalizability of previously published research on this issue was problematic. To the best of our knowledge, the lack of exploration of the link between organizational trust and job satisfaction among NPs remains a gap in the literature.

In considering the results of previous literature, we applied Herzberg's motivation-hygiene theory to explore potential factors affecting job satisfaction and team-based practice among NPs. Herzberg [37] classified factors influencing job satisfaction into motivators and hygiene factors in the workplace. The motivators are sometimes referred to as satisfiers, which are intrinsic conditions of the job itself, such as personal growth, opportunities for advancement, and a sense of importance to an organization. The hygiene factors are sometimes referred to as dissatisfiers, which are extrinsic to the work itself, such as working conditions, relationships with colleagues or supervisors, and organizational policies. If these hygiene factors were absent, staff would demonstrate more dissatisfaction with their job. In the current study, we classified NPs' demographic characteristics, organizational culture, and organizational trust as motivators or hygiene factors. We aimed and hypothesized that various organizational cultures and organizational trust affect NPs' job satisfaction and team-based practice.

## 2. Methods

### 2.1. Design

This study had a cross-sectional design, and it involved a national online survey to explore potential factors influencing job satisfaction and team-based practice.

### 2.2. Participants

We recruited participants from the Taiwan Association of Nurse Practitioners (TANP). NPs who had national certified licenses and had been working as NPs in acute care settings for at least 1 year were eligible for inclusion.

### 2.3. Ethical Considerations

The study was approved by the Institutional Review Board (IRB) of the China Medical University Hospital (CMUH111–REC3–059). Before eligible NPs filled out the questionnaire, they were asked to read the detailed information about the contents of the study and complete a written informed consent via a unique link to the online survey. The study was also conducted in compliance with the ethical standards of the Helsinki Declaration.

### 2.4. Data Collection

There were 9,536 active members of TANP, and 6,808 eligible NPs were invited to participate from March to May 2022. Detailed information about the study's aims, procedures, written informed consent, and a unique link to the online survey were provided in each e-mail. In total, 1,113 NPs agreed to participate and completed the survey. The response rate was 16.35%. Thirteen participants were excluded from the analysis due to incomplete data (e.g., missing values in demographic data). As result, 1,100 participants were retained for further analysis. There was no significant difference between incomplete data and analyzed data in job satisfaction (*t* = 0.07, 95% CI (−2.60, 2.80), *p*=0.94) and teamwork (*t* < 0.001, 95% CI (−0.25, 0.25), *p*=1.00). The setting of a power analysis was conducted with *G* *∗* Power 3.1.9.7 for multiple regression analysis [38, 39]. According to the previous finding, the effect size (Cohen's *f*^2^) ranged from 0.19 to 0.53 [15, 25]. We used a medium effect size value of 0.15 to conservatively calculate the sample size. With the effect size (Cohen's *f*^2^) of 0.15, a statistical power of 0.90, a significant level of 0.05, and 10 potential predictors, the estimated sample size was 147. In addition, we assumed that the attrition rate of the online survey was about 10% [40]. Hence, the effective sample size for multiple regression analysis should be more than 162. The final number of the current study (*n* = 1,100) was sufficient to conduct the analysis.

### 2.5. Measurement

#### 2.5.1. Demographic and Working Characteristics

The demographic characteristics of NPs, including age, gender, marital status, educational degree, working status, hospital level of practice, working hours per day, years of NP experience, NP advancement level, patient load, and annual salary, were surveyed by a series of questions.

#### 2.5.2. Job Satisfaction

NPs' job satisfaction was assessed using the Misener Nurse Practitioner Job Satisfaction Scale (MNPJSS) developed by Misener and Cox [41]. This scale comprised 44 self-administered items with six dimensions, namely intrapractice partnership/collegiality; challenge/autonomy; professional, social, and community interaction; professional growth; time; and benefits. Each item was rated on a 6-point scale (ranging from very dissatisfied to very satisfied). Higher scores indicated a higher level of job satisfaction perceived by individuals in practice. MNPJSS has demonstrated proper reliability and validity in previous research relating to job satisfaction, and Cronbach's alpha was 0.94–0.96 [41, 42]. Cronbach's alpha value for this study was 0.95.

#### 2.5.3. Team-Based Care

NPs' team-based care was evaluated using the NP-physician relations (NP-PR) subscale of the Nurse Practitioner Primary Care Organizational Climate (NP-PCOCQ) developed by Poghosyan et al. [43]. This scale was developed specifically for the NP practice environment. This scale comprised 29 self-administered items with four subscales: NP-PR, professional visibility (PV), NP-administration relations (NP-AR), and independent practice and support (IPS). Each item was rated on a 4-point scale (ranging from strongly disagree to strongly agree). The NP-PR subscale measured the teamwork, communication, and relationships between NPs and physicians in practice. Higher scores obtained from the NP-PR subscale indicated better teamwork in practice. Previous research also showed adequate reliability and validity. The Cronbach alpha of this subscale ranged from 0.87 to 0.95 in previous studies [43, 44]. Cronbach's alpha value for the NP-PR subscale for this study was 0.90.

#### 2.5.4. Organizational Culture

NPs' organizational culture was measured with the Organizational Culture Scale designed by Bradley et al. [45]. This scale could identify substantial diversity in culture related to patient care. It consisted of 31 items rated on a 5-point scale with options from 1 (disagree strongly) to 5 (agree strongly). This scale detected organizational culture in five domains: (a) learning environment, (b) psychological safety, (c) commitment to the organization, (d) senior leadership support, and (e) time for improvement efforts. The learning environment indicated that hospitals provided the latest information or knowledge about patient care for NPs. Psychological safety revealed that hospitals valued NPs' challenging assumptions, new ideas, or unique skills, and that it is quite easy to seek help in practice. Commitment to the organization indicated the emotional attachment between NPs and hospitals. Senior leadership support occurred when opinion leaders focused on improvement, encouraged changes in the quality of patient care, and offered the necessary resources (e.g., personnel or equipment) for NPs in practice. Time for improvement efforts refers to the time invested in enhancing the quality of patients' care. Higher scores indicated that NPs perceived a higher level of organizational culture in the hospital. This Cronbach's alpha was 0.94 for the entire scale and ranged from 0.77 to 0.88 for the subscales, which showed substantial reliability and validity in previous studies [45, 46]. The Cronbach alpha was 0.91 for the whole scale, and it ranged from 0.61 to 0.93 for the subscales (learning = 0.93; psychological safety = 0.78; commitment = 0.69; support = 0.87; time for improvement = 0.61).

#### 2.5.5. Organizational Trust

Organizational trust was assessed using the scale developed by Gabarro and Athos [47]. This scale is a 5-item self-reported scale rated on a 4-point Likert-type scale from 1 (strongly disagree) to 4 (strongly agree). Higher scores indicate a stronger level of employee trust in the organization. Previous research also demonstrated satisfactory reliability and validity for this scale and Cronbach's alpha was 0.94 [47, 48]. Cronbach's alpha in the current study was 0.82.

### 2.6. Statistical Analysis

The distributions of NPs' characteristics and study variables were used for univariate descriptive analyses including percentage, mean, and standard deviation (SD). An independent *t*-test was used to compare the means of two independent groups. Pearson product-moment correlation coefficients (*γ*) were calculated to examine the correlations between two continuous variables. Potential factors influencing job satisfaction and team-based practice were explored by multiple regression analysis with a stepwise selection method. The stepwise selection aims to identify a subset of variables that are most relevant for predicting job satisfaction. The significant variables from the univariate analysis were included in the regression analysis. The unstandardized coefficient (*B*), 95% confidence interval (95% CI), standardized coefficient (*β*), standard error (SE), Δ*R*^2^, and adjusted *R*^2^ were also estimated by the multiple regression model. The multicollinearity of regression was examined with the variance inflation factor (VIF) [49]. The VIF should be less than 5 [50]. All data were analyzed with SPSS Statistics Version 22.0 (IBM, Armonk, NY, USA) software. The value of significance was 0.05.

## 3. Results

### 3.1. Descriptive Statistics for NPs' Characteristics

A total of 1,100 NPs with completed data were included in statistical analyses. The average age of our sample was 43.49 (SD = 5.84). Most NPs were females (*n* = 1.052; 95.60%), married (*n* = 729, 66.30%), and had completed a university (*n* = 896; 81.50%) or Master's/Ph.D. degree (*n* = 204, 18.5%). The average years of experience as NPs were 9.12 years (SD = 4.10), and most worked in regional hospitals (*n* = 776; 70.50%). More than half of NPs worked fixed shifts (*n* = 619; 56.30%), the average daily working hours were 9.16 (SD = 1.40), and they cared for 13.3 (SD = 8.39) patients per day shift. The NPs' professional career ladder level was mainly NP1 (*n* = 271, 24.6%) or NP2 (*n* = 411, 37.4%) (Table 1).

### 3.2. Correlations between NPs' Background, Job Satisfaction, and Team-Based Practice

The correlations between NPs demographic characteristics, job satisfaction, and team-based practice are summarised in Table 2. NPs who were married (*t* = −2.36, 95% CI (−0.81, −8.88), *p*=0.02), higher on the professional career ladder level (*r* = 0.12, *p* < 0.001), and had higher annual salaries (*r* = 0.15, *p* < 0.001) reported higher job satisfaction. Daily working hours were negatively related to job satisfaction (*r* = −0.09, *p* < 0.01). Age (*r* = 0.08, *p*=0.01), years of NP experience (*r* = 0.11, *p* < 0.001), professional career ladder level (*r* = 0.09, *p* < 0.01), and annual salary (*r* = 0.12, *p* < 0.001) were positively correlated with team-based practice. However, working in a rotating shift rather than on a fixed shift showed a lower level of team-based practice (*t* = −2.89, 95% CI (−0.17, −0.87), *p* < 0.01).

There was a significant positive association between job satisfaction and organizational trust (*r* = 0.55, *p* < 0.001) (Table 3). Job satisfaction was positively associated with all dimensions of organizational culture (*r* ranged from 0.56 to 0.62, *p* < 0.001) except for time for improvement. Furthermore, team-based practice was positively associated with organizational trust (*r* = 0.45, *p* < 0.001). Except for the time for improvement, all subscales of organizational culture were positively associated with team-based practice (*r* ranged from 0.31 to 0.39, *p* < 0.001).

### 3.3. Factors Influencing Job Satisfaction and Team-Based Practice

The results of the stepwise multiple regression on NPs' job satisfaction are in Table 4. Some 49.2% of the variances in NPs' job satisfaction were explained by organizational culture (learning environment, commitment to the organization, psychological safety, and senior leadership support), and organizational trust. Of these, the primary two variables that accounted for 37.8% of the variances were the learning environment dimension of organizational culture and psychological safety (7.2%). The higher learning environment (*B* = 1.18, 95% CI (0.77, 1.59), *p* < 0.001), psychological safety (*B* = 1.77, 95% CI (1.34, 2.21), *p* < 0.001), senior leadership support (*B* = 1.54, 95% CI (0.85, 2.23), *p* < 0.001), commitment to the organization (*B* = 0.81, 95% CI (0.33, 1.30), *p* < 0.01), and organizational trust (*B* = 2.59, 95% CI (1.75, 3.42), *p* < 0.001) were positively associated with higher job satisfaction.

Organizational trust, organizational culture (commitment to the organization and learning environment), and working status accounted for 23.66% of the variance in NPs' team-based practice. Organizational trust was the strongest independent predictor accounting for 20% of the variance in terms of team-based practice. Higher organizational trust (*B* = 0.37, 95% CI (0.27, 0.46), *p* < 0.001), commitment to the organization (*B* = 0.13, 95% CI (0.08, 0.18), *p* < 0.001), and learning environment (*B* = 0.07, 95% CI (0.13, 0.11), *p*=0.001) were positively associated with higher team-based practice. Moreover, NPs who worked fixed shifts rather than rotating shifts had higher levels of team-based practice (*B* = 0.38, 95% CI (0.07, 0.69), *p* < 0.001) (Table 4).

The VIFs of each significant predictor for job satisfaction (which ranged from 1.82 to 2.74) and team-based practice (which ranged from 1.00 to 1.92) were less than 5, which indicated that there was no multicollinearity in the multiple regression model [50].

## 4. Discussion

Our findings were consistent with Herzberg's motivation-hygiene theory. We found that motivators such as organizational trust could promote both job satisfaction and team-based practice in practice. For the hygiene factors, only working status reduced NPs' performance in team-based practice. These results highlight the importance of the organizations' role in significantly improving the positive organizational culture in healthcare organizations and trust levels within the practice. Moreover, managers or supervisors could modify the working shift patterns of NPs to ensure better team-based practice.

### 4.1. Variables Affecting Job Satisfaction

**Figure 1:**
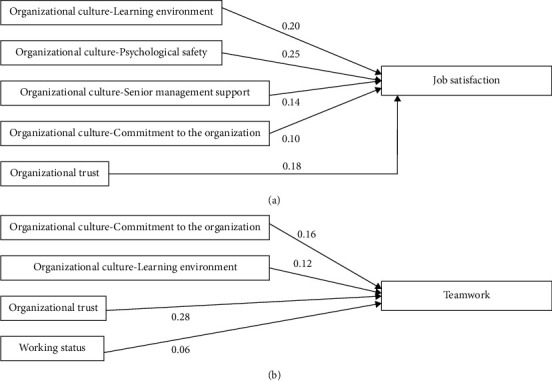
Effect of variables on job satisfaction and teamwork.

Our study identified specific dimensions of organizational culture that influenced NPs' job satisfaction, which was consistent with Herzberg's motivation-hygiene theory (Figure 1(a)). To begin with, better learning environments improve job satisfaction. This might be because the learning environment may inspire NPs to put forth the remarkable effort and enhance their professional competency and professional growth. Along similar lines, hospitals with better learning environments could help NPs to gain new knowledge and advanced skills in the process of providing care. The internally motivated work behavior could improve the psychological states of employees (e.g., job satisfaction in the current study) [51]. One recent study also indicated that the utilization of NPs' education or on-the-job training was significantly correlated with job satisfaction [52]. The learning environment might increase practice variety and enhance NPs' motivation while producing better effectiveness in practice. Laschinger et al. [53] indicated that the enhancement of professional knowledge and expertise is critical for being effective in practice. Similarly, NPs viewed learning environments such as by continuing education as a vital element for professional growth, which could be recognized for their organizational efforts and enhance job satisfaction [54]. Subsequently, the learning environment increases NPs' identification and effectiveness of practice, which promote job satisfaction. To improve job satisfaction, the healthcare organizations should develop a supportive learning environment at the organizational level such as implementing advanced education programs, enhancing interdisciplinary communication, and improving the process of care by utilizing the latest information or clinical equipment.

Furthermore, our study also found that senior management support can increase NPs' job satisfaction, which is supported by previous studies [8, 10]. Senior managers could gain greater insight and provide the necessary support or resources for NPs to deliver care, thereby increasing NPs' job satisfaction. Poghosyan et al. [10] noted that the importance of organizational support, especially higher organizational-level support, was associated with higher job satisfaction. Eskandari et al. [55] also indicated that nursing leaders or managers were key in creating supportive working environments, which increases job satisfaction. Thus, support from senior managers is essential for the job satisfaction of NPs. On the other hand, senior managers could also implement policies to empower NPs' practice. Senior managers could coordinate with other supervisors or middle managers and even convince administrators that sufficient empowerment is critical for acute care and that it can improve job satisfaction. Consequently, NPs could practice more efficiently and report better job satisfaction. This finding suggests that it is necessary to encourage NPs to communicate with senior managers and that senior managers should be proactive in empowering NPs at the organizational level of practice by implementing policies to improve job satisfaction.

Moreover, we found that feelings of psychological safety could increase job satisfaction. Psychological safety could be defined as “an individual's perceptions of the consequences of taking interpersonal risks in a workplace” [56], P. 23). The positive effect of psychological safety on job satisfaction might likely contribute to sufficient support from organizations. The NP system was implemented for over fifteen years in Taiwan, and hospital administrators aware of the scope of practice of NPs and have given them the necessary support for practice [5]. In consequence, NPs perceived greater psychological safety and improved job satisfaction. Therefore, healthcare organizations should continue to support NPs' practice at the organizational level.

Lastly, our result showed that the greater organization's commitment, the better job satisfaction was demonstrated. This finding was supported by previous studies documenting that commitment was a significant predictor of job satisfaction [6, 30]. This would be explained that job satisfaction would increase by the greater organizations' commitment through satisfactory empowerment. According to Meyer and Allen [57], organizational commitment refers to psychological attachment or affective commitment initiated in relation to an individual's identification and involvement within the organization. NPs with supportive working environments might fully practice their professional role which promotes organizational commitment. Moreover, recent studies had showed that organizational support is a vital factor in enhancing work commitment [58, 59]. Therefore, healthcare organizations are suggested to modify policies to balance the practice rights of NPs and physicians in the collaborative practice model, which might strengthen the organizational commitment and improve job satisfaction of NPs.

Except for organizational culture, the results of our study have revealed the positive effect of organizational trust on job satisfaction. This might be explained by different authorizations of hospitals to allow NP practice [9, 60]. This lack of uniform guidelines of practice may cause conflicts between NPs' professional roles and hospitals' regulations, which could lead to limits on NPs' practice rights. Thus, NPs might not trust hospitals and might therefore have poor job satisfaction [61]. On the contrary, a sufficient organizational trust might enable NPs to perceive their own professional value and enhance job satisfaction [34]. The health department of the government should formulate uniform guidelines of practice to promote the trust relationship between NPs and organizations; hence, NPs would show better job satisfaction.

### 4.2. Variables Affecting Team-Based Practice

The current study showed that team-based practice was influenced by organizational trust, organizational culture, and the fixed shifts (Figure 1(b)). Above all, organizational trust is essential to improve team-based practice, which was in accordance with previous studies [33, 35, 36]. This might be because organizational trust could improve communication between team members [28, 29]. In Taiwan, NPs mainly work with physicians to deliver team-based care to meet the complex needs of patients [44]; hence, effective communication is vital for team-based care. Ozluk and Baykal [62] noted that healthcare professionals provide care for patients, which requires high concentration in the collaboration process, so even the smallest neglect might lead to patient safety issues. Employees are likely to reciprocate organizational trust through better performance as organizations provide employees with sufficient trust from managers (vertical trust) or coworkers [35]. Consequently, NPs would perceive more organizational trust, which may lead to better team-based practice.

Furthermore, our findings demonstrated that team-based practice was positively influenced by the learning environment. The practice of NPs is complex and improvements in the learning environment such as new skills or sharing information, along with better cooperation with other professional staff are critical for healthcare. A previous review study also noted that there would be beneficial effects for health professionals to experience good learning environments such as teamwork education programs in acute care settings [63]. If organizations focused on building the culture including continuous improvement in the practice, it would encourage NPs to deliver superior performance [64]. Similarly, Engle et al. [65] found that better organizational cultures had links with high performance, and they indicated that some medical centers fostered multidisciplinary approaches, which could facilitate the delivery of both evidence-based practice and patient care. Therefore, they suggested that organizations should enhance the opportunities for interdisciplinary collaboration and the implementation of digital data management to increase the performance of team-based practice in acute care settings.

In addition, our findings also indicated that organizational commitment could increase the effectiveness of team-based practice. We found that organizational culture had direct effects on team-based practice, which were comparable with previous studies [22, 23, 25]. This might be explained by the fact that NPs' roles were ambiguous in the collaborative care model as they might not be adequately supported. Even though NPs worked with physicians to deliver team-based care and basically followed practice guidelines. Therefore, organizations need to empower NPs with policies to ensure their clinical practice is valued within team-based care and each team member is aligned with his or her responsibility in patient care, which promotes efficient team-based practice.

Lastly, we found that NPs were more likely to demonstrate high efficiency in team-based practice, as they worked on a fixed schedule, which was consistent with previous studies [66, 67]. This result might contribute to the distress or burnout shift workers experience. Compared to workers with fixed schedules, they reported more frequent burnout, which also leads to circadian rhythm disruption [68]. The rotating shift workers had insufficient rest time, which led to burnout or fatigue [69]. As a result, teamwork care may be compromised. Furthermore, NPs with rotating shift schedules may have to collaborate with many healthcare teams in practice, and this can reduce job satisfaction [70]. Hence, on a fixed working schedule, it is easier to develop positive relationships between team members and better team-based practice. Healthcare organizations are encouraged to schedule NPs on fixed shifts to alleviate the impact of shift work on team-based care.

### 4.3. Limitations

This study has some limitations. First, as this study had a cross-sectional design, it was not possible to make causal inferences between organizational culture and organizational trust with job satisfaction and teamwork in practice. Nevertheless, our results may be viewed as exploratory, and other researchers might identify distinct motivators or hygiene factors in organizational contexts affecting job satisfaction or teamwork over time. Furthermore, the participants in this study were only recruited from Taiwan, and organizational culture could vary in different countries. Hence, the generalizability of our findings could be limited. The culture difference might also lead to a lower Cronbach's alpha for time for improvement (Cronbach's alpha = 0.61) in the current study. The contents of this subscale might modify for better reliability in future studies. In addition, we used the subscale of NP-PCOCQ to measure teamwork, and it was not designed to be a standalone tool to measure teamwork. However, patients' healthcare mainly relied on the collaboration between NPs and physicians in Taiwan, such collaboration would reflect the performance of teamwork. Future studies would conduct a unique tool for measuring teamwork in studies. Finally, there might be a response bias due to the self-completed questionnaire design. Despite these limitations, this study has uncovered the influence of organizational context on both job satisfaction and team-based practice using a national-based survey in Taiwan.

## 5. Conclusion

Organizational culture and trust were key for improvements in job satisfaction and team-based practice. The learning environment and organizational trust were the two most influential factors in job satisfaction and team-based practice, respectively. Hospitals could establish a supportive atmosphere of practice using an appropriate organizational culture and providing sufficient trust to promote both NPs' job satisfaction and team-based practice. Also, because the effects of organizational culture and trust on job satisfaction and team-based practice have not been studied previously in a national sample, our results provide a foundation to fill the knowledge gap in this field and to develop future studies.

## 6. Implication for Nursing Management

Continuing to improve the organizational culture and trust of NPs is critical to enhancing NPs' job satisfaction and teamwork in acute care practices. In the process, hospital administrators are encouraged to develop and implement policies to enrich supportive organizational cultures, such as enhancing the learning environment and building up an interdisciplinary collaboration that could improve job satisfaction and team-based care. Moreover, empowering NPs in practice at the organizational level and encouraging communication with senior managers are beneficial for increasing NPs' psychological safety and gaining the support of senior managers.

It is also suggested that acute care hospitals need to implement policies to ensure the rights of NPs in the collaborative practice model, which therefore might improve job satisfaction and efficiency of the team-based practice. In particular, authorized practice guidelines might strengthen organizational trust in acute care settings, which promotes better job satisfaction and proficient team-based practice.

## Figures and Tables

**Table 1:** NPs' characteristics and study variables (*N* = 1,100).

Variable	*n* (mean)	% (SD)
Age (years)	43.49	5.84
*Gender*		
Female	1,052	95.60
Male	48	4.40
*Marital status*		
Married	729	66.30
Unmarried	371	33.70
*Educational degree*		
University	896	81.50
Graduate school or above	204	18.50
*Working status*		
Fixed shift	619	56.30
Rotating shift	481	43.70
*Hospital level*		
Medical center	324	29.50
Regional hospital	776	70.50
*Professional career ladder*		
NP1	271	24.6
NP2	411	37.4
NP3	118	10.7
NP4	17	1.5
NP5	41	3.7
Daily working hours	9.16	1.40
NP experience (years)	9.12	4.10
Daily patient load	13.38	8.39
Organizational trust	14.96	2.27
*Organizational culture*		
Learning environment	28.66	5.54
Psychological safety	25.62	4.46
Commitment to the organization	22.36	3.82
Senior management support	13.64	2.85
Time for improvement efforts	11.90	2.36
MNPJSS (job satisfaction)	168.51	32.31
Team-based practice	21.54	2.98

*Note*. SD, standard deviation; MNPJSS, Misener Nurse Practitioner Job Satisfaction Scale.

**Table 2:** Correlations between demographic and work characteristics, job satisfaction, and team-based practice (*N* = 1,100).

Variable	Job satisfaction				Team-based practice			
	Mean	SD	*t*/*r*	*p*	Mean	SD	*t*/*r*	*p*
Age (years)	—	—	0.56	0.07	—	—	0.08^*∗*^	0.01
*Gender*								
Female	168.31	32.12	−0.96	0.34	21.54	2.93	−0.08	0.94
Male	172.90	36.36			21.58	3.92		
*Marital status*								
Married	170.14	30.94	−2.36^*∗*^	0.02	21.60	2.99	−0.93	0.35
Unmarried	165.30	34.67			21.42	2.97		
*Educational degree*								
University	168.68	31.45	0.35	0.73	21.49	2.89	−1.06	0.29
Graduate school or above	167.74	35.93			21.76	3.36		
*Working status*								
Fixed shift	169.09	31.85	−0.68	0.50	21.77	3.04	−2.89^*∗∗*^	0.004
Rotating shift	167.75	32.91			21.25	2.88		
*Hospital level*								
Medical center	169.19	32.53	1.08	0.28	21.34	2.77	1.51	0.13
Regional hospital	166.88	31.78			21.63	3.06		
Daily working hours	—	—	−0.09^*∗∗*^	0.002	—	—	−0.03	0.34
NP experience (years)	—	—	0.06	0.07	—	—	0.11^*∗∗∗*^	<0.001
Professional career ladder	—	—	0.12^*∗∗∗*^	<0.001	—	—	0.09^*∗∗*^	<0.01
Daily patient load	—	—	−0.03	0.27	—	—	−0.002	0.93
Annual salary	—	—	0.15^*∗∗∗*^	<0.001	—	—	0.12^*∗∗∗*^	<0.001

*Note*. ^*∗*^*p* < 0.05; ^*∗∗*^*p* < 0.01; ^*∗∗∗*^*p* < 0.001.

**Table 3:** Correlations among organizational culture, organizational trust, job satisfaction, and team-based practice (*N* = 1,100).

Variable	Job satisfaction	Team-based practice
Organizational trust	0.55^*∗∗∗*^	0.45^*∗∗∗*^
*Organizational culture*		
Learning environment	0.62^*∗∗∗*^	0.39^*∗∗∗*^
Psychological safety	0.59^*∗∗∗*^	0.34^*∗∗∗*^
Commitment to organization	0.51^*∗∗∗*^	0.37^*∗∗∗*^
Senior management support	0.56^*∗∗∗*^	0.31^*∗∗∗*^
Time for improvement efforts	0.02	−0.01
Total	0.69^*∗∗∗*^	0.42^*∗∗∗*^

*Note*. ^*∗∗∗*^*p* < 0.001.

**Table 4:** Potential factors associated with job satisfaction and teamwork among acute care NPs (*N* = 1,100).

Variables	*B*	SE	95% CI^a^	*β*	*t*	*p*	Δ*R*^2^
*Job satisfaction*							
Constant	11.37	5.23	4.87, 25.05	—	2.17	0.03^*∗*^	
*Organizational culture*							
Learning environment	1.18	0.21	0.77, 1.59	0.20	5.68	<0.001^*∗∗∗*^	0.378
Psychological safety	1.77	0.22	1.34, 2.21	0.25	8.02	<0.001^*∗∗∗*^	0.072
Organizational trust	2.59	0.43	1.75, 3.42	0.18	6.06	<0.001^*∗∗∗*^	0.027
*Organizational culture*							
Senior management support	1.54	0.35	0.85, 2.23	0.14	4.38	<0.001^*∗∗∗*^	0.01
Commitment to the organization	0.81	0.25	0.33, 1.30	0.10	3.31	<0.01^*∗∗*^	0.005
Adjusted *R*^2^							0.492

*Teamwork*							
Constant	11.11	0.57	9.98, 12.24	—	19.35	<0.001^*∗∗∗*^	
Organizational trust	0.37	0.05	0.27, 0.46	0.28	7.61	<0.001^*∗∗∗*^	0.20
*Organizational culture*							
Commitment to the organization	0.13	0.03	0.08, 0.18	0.16	5.09	<0.001^*∗∗∗*^	0.027
Learning environment	0.07	0.02	0.03, 0.11	0.12	3.37	0.001^*∗∗*^	0.008
Working status (ref: shift)	0.38	0.16	0.07, 0.69	0.06	2.41	0.02^*∗*^	0.004
Adjusted *R*^2^							0.236

*Note*. ^*∗*^*p* < 0.05; ^*∗∗*^*p* < 0.01; ^*∗∗∗*^*p* < 0.001. ^a^The 95% CI was estimated by the unstandardized coefficient (*B*).

## Data Availability

The data used to support the findings of this study are available on request from the author.
